# Micro to nano: Surface size scale and superhydrophobicity

**DOI:** 10.3762/bjnano.2.38

**Published:** 2011-06-27

**Authors:** Christian Dorrer, Jürgen Rühe

**Affiliations:** 1 University of Freiburg, Department of Microsystems Engineering, Laboratory for the Chemistry and Physics of Interfaces, Georges-Köhler-Allee 103, D-79110 Freiburg, Germany

**Keywords:** contact angle, hysteresis, superhydrophobic, wetting

## Abstract

This work looks at the fundamental question of how the surface mobility of drops in the composite state is related to the size scale of the roughness features of the surface. To this end, relevant literature is first reviewed and the important terms are clarified. We then describe and discuss contact and roll-off angle measurements on a set of hydrophobicized silicon post surfaces for which all parameters except for the surface size scale were held constant. It was found that a critical transition from “sticky superhydrophobic” (composite state with large contact angle hysteresis) to “truly superhydrophobic” (composite state with low hysteresis) takes place as the size of the surface features reaches 1 μm.

## Introduction

**Figure 1:**
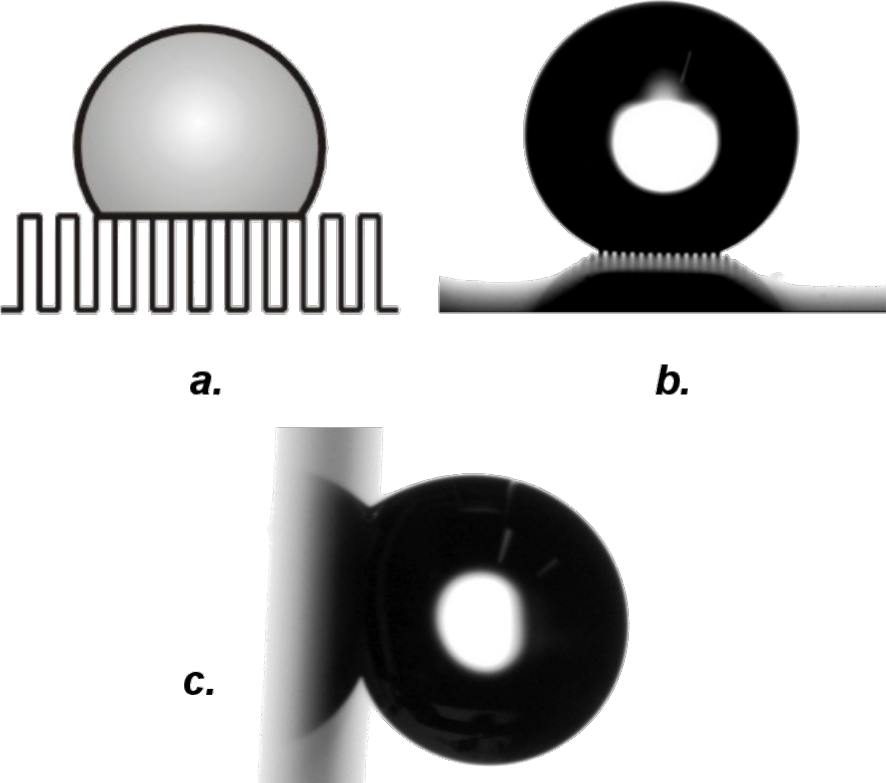
Composite wetting: Drops are suspended on top of the roughness features, with air trapped underneath. The drops are resting on a carpet composed of solid and air; the term “composite” is therefore used. (a) Schematic depiction and (b) micrograph of a drop on a microscale post surface. The width of the posts was 16 μm. (c) The surface from b has been rotated by 90°. Even though the drop was in the composite state, the hysteresis was considerable, as witnessed by the fact that the drop stuck to the surface even at extreme tilting angles.

Superhydrophobic surfaces have recently been the focus of considerable scientific interest [1–10]. This is due to the fact that artificial superhydrophobic surfaces are promising candidates for a number of practical applications, for example, self-cleaning windows, clothing, and also microfludic systems. Drops that come into contact with a superhydrophobic material retain a nearly spherical shape and can easily roll off. As has been shown, this effect results from a wetting situation (referred to as *Cassie* or *composite* wetting) where liquids no longer penetrate, but rest on top of the roughness features [1–2,11]. Air remains enclosed underneath, and drops are therefore supported by a “composite surface” that consists of solid and air (Figure 1a and 1b). For this situation, Cassie’s original theory computes the contact angle (CA) of a drop from the CA on the smooth material, θ_S_, and the fraction of the drop footprint in contact with the solid, the solid fraction 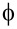, according to [11]:

[1]
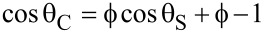


The contact line samples the different components of the composite surface, averaging to form the macroscopic CA. Equation 1 is therefore an approximation that becomes better as the size of the surface features decreases relative to the drop size [12].

On most surfaces, the motion of the liquid meniscus is opposed by energy barriers, which leads to an effect referred to as contact angle hysteresis [2,12–13]. As a result of CA hysteresis, the actual CA of a composite drop (this term is loosely used to denote a drop that is in the composite state) may deviate from the value predicted by Cassie’s theory, with the maximum/minimum angle appearing for a liquid front that is being advanced/receded over the substrate (these angles are then referred to as the advancing/receding contact angles) [12–13]. With respect to the composite state, the precise nature of the energy barriers that govern the movement of drops has remained unclear, although recent research seems to suggest that, in particular, events taking place on the receding portions of the contact line play an important role [3–4,14]. Superhydrophobic wetting can be considered a special case of composite wetting. On a superhydrophobic material, the energy barriers associated with the motion of drops are extremely small, and the CA hysteresis, Δθ, is close to zero [1–10]. As a result, water drops remain extremely mobile and roll off even if only very small forces are applied, for example by slightly tilting the substrates. In the course of this process, the drops may pick up dirt particles, effectively leading to a “self-cleaning” of the respective interface.

It is important to realize that not all surfaces for which composite wetting is observed are superhydrophobic: The hysteresis on purely microstructured, hydrophobicized post surfaces, for instance, often remains considerable even though quite high static CAs are sometimes measured [14–16]. In one example from the literature, a contact angle hysteresis of 14° was measured for a surface equipped with hydrophobicized posts 8 μm wide and spaced at 32 μm [15]. Recent results from our laboratory revealed a CA hysteresis of 12° for a hydrophobic post surface where the post width was 4 μm and the post spacing 16 μm [14]. While drops on the latter surface indeed appeared spherical due the relatively high static contact angles (around 165°), in particular, small drops of the size commonly used for contact angle measurements (around 2 μL) were not very mobile, requiring considerable tilting angles (>30°) to roll off. Especially when compared to other materials where even smallest drops roll off at tilting angles of only 5° or less (see, for example, the superhydrophobic surfaces from [2–9]), this post surface can not be called “truly” superhydrophobic; indeed, the oxymoron “sticky superhydrophobic” coined by Gao et al. [17] is more appropriate. The fact that composite wetting does not equal superhydrophobicity is illustrated in Figure 1c: In the experiment that is shown, a tilting angle of 90° was not sufficient to dislodge a composite drop resting on a hydrophobicized post surface where the posts were 16 μm wide and spaced at 16 μm.

What makes a surface “truly” superhydrophobic? Recent research seems to suggest that, firstly, the surface size scale also plays a role [18]. For example, it was observed that the lotus leaf looses its water-repellent properties if the source of the nanoscale roughness, which originates from fine hairs with a diameter of a few nanometers, is removed [19]. In addition, hydrophobicized, microscale surfaces with posts 4 μm wide become “truly” superhydrophobic if the post tops are equipped with an additional nanostructure [3]. An important question is: How small do the surface features need to be in order to induce superhydrophobic properties? Secondly, it has been found that the shape of the roughness features has an influence: For instance, square shaped posts behave differently from star-shaped ones [15]. And thirdly, it has been speculated that the solid fraction parameter 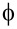 from Equation 1 needs to be considered, in the sense that a lower solid fraction leads to a lowered hysteresis [20–21]. Answering these points is not possible from the experimental data that is currently available, because the different experimental series that have been conducted in different laboratories always differ in more than one parameter. For example, hydrophobicized post surfaces differ from a fibrous superhydrophobic surface such as the one presented in [9], both in size scale and shape of the roughness features. The nanoscale posts investigated by Martines et al. differ from larger-scale post surfaces both in surface chemistry and post shape [22]. In the present work, surfaces were created that spanned different size scales, but remained constant with respect to solid fraction, surface chemistry and the shape of the surface features. From the characterization of these surfaces, conclusions were drawn with respect to the effect of the surface size scale on the behavior of drops in the composite state.

## Results and Discussion

**Figure 2:**
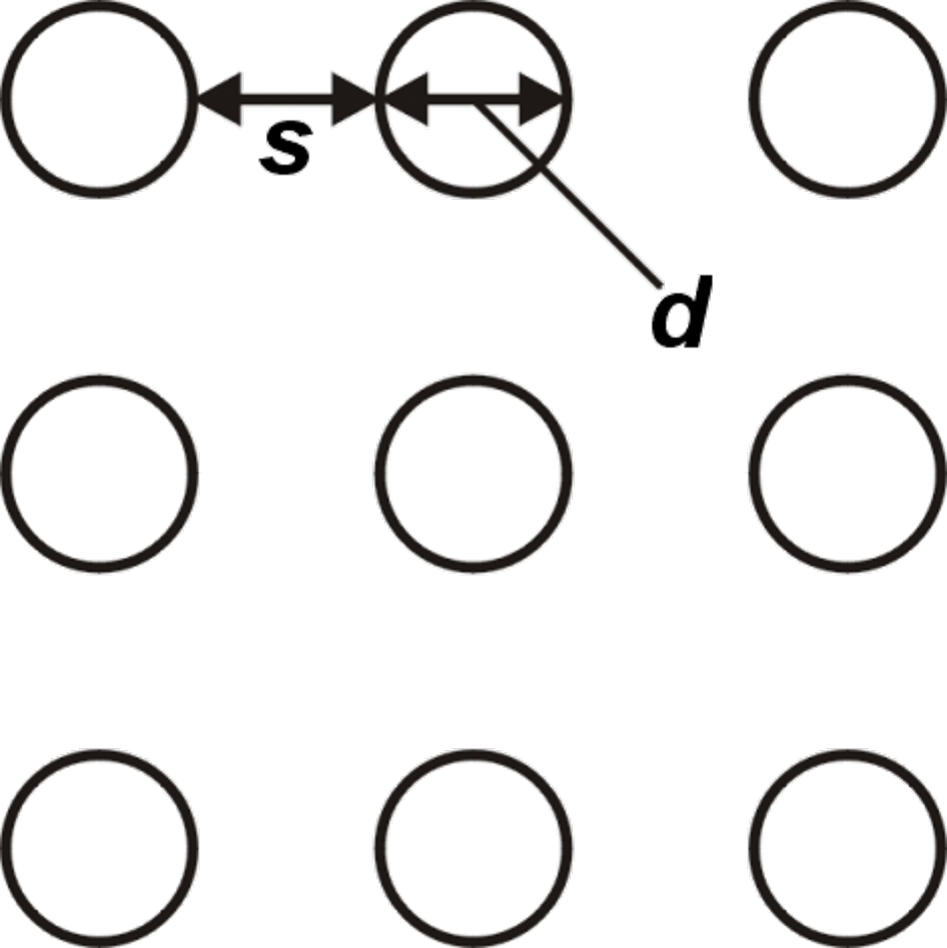
Geometry of the post surfaces with *d* = post width and *s* = post spacing.

**Figure 3:**
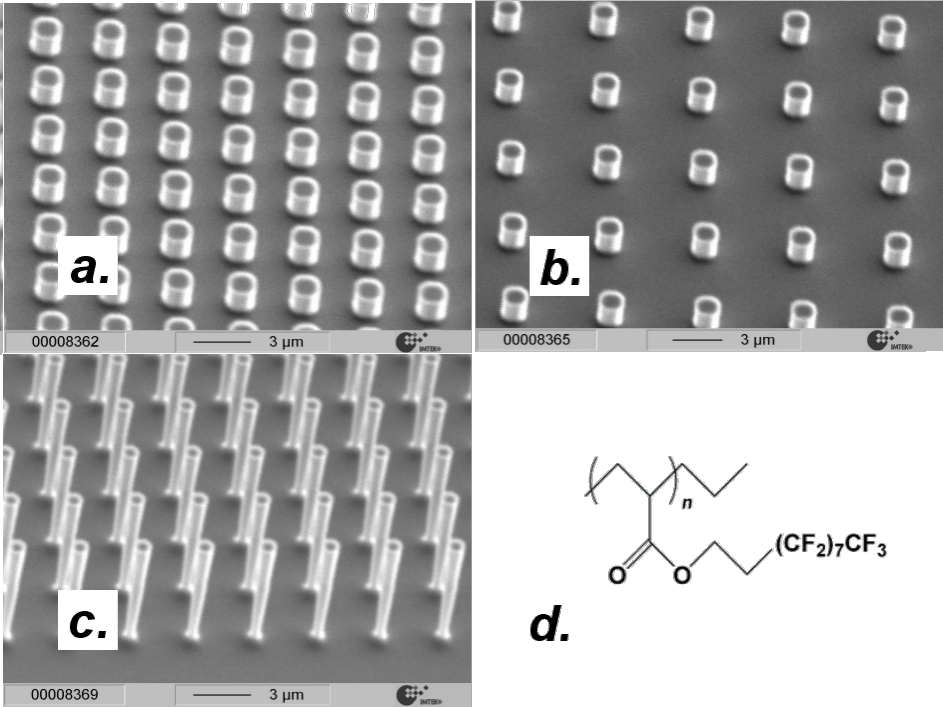
SEM-images of the micromachined silicon post surfaces. (a) Surface with *d* = *s* = 1 μm, (b) surface with *d* = 1 μm, *s* = 4 μm. (c) Lateral dimensions as for b, post height = 8 μm. (d) Chemical formula of the polymer that was used for hydrophobization.

In two recent publications, we have reported on the characterization of silicon post surfaces that, once coated with a thin layer of a hydrophobic fluoropolymer, were wetted in the composite mode [14,23]. For such hydrophobicized post-type materials, the solid fraction is easily derived from the top area of the posts because, under normal circumstances (i.e., without the application of an additional volume force), the liquid menscus is unable to penetrate the post structure. In this work, following identical experimental procedures as described previously [14,22–24], we have generated three sets of hydrophobicized silicon post surfaces through lithography and micromachining techniques. Our goal in these experiments was to systematically vary the surface size scale. In order to isolate the effect of this parameter on the wetting properties, other geometrical parameters such as the solid fraction 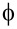 were held constant. The grid on which the posts were arranged was quadratic (as in Figure 2). The following geometries were generated: *Series a*: Post width *d* = 16, 8, 4, 1.5, 1.2 μm, post spacing *s* = *d*. *Series b*: *d* = 10, 5, 1, 0.5 μm, *s* = 2*d*. *Series c*: *d* = 10, 5, 1.3, 1, 0.5 μm, *s* = 4*d*. The three series corresponded to solid fractions of 25 ± 2% (series a), 11 ± 2% (series b) and 4 ± 1% (series c) (the solid fractions were verified by extracting the top area of the posts from the SEM images). Figure 3 shows a selection of SEM images, together with the chemical formula of the polymer that was used for hydrophobization. The shape of the post cross-sections was circular. The post shapes were the same for all posts; thereby, any effect of this parameter on the wetting behavior could be excluded. For all series, the smaller posts where 2 μm and the larger posts 8 μm tall. Since the drops rested on top of the structure, the post height (or the fact that the taller posts were tapered) had no effect on the wetting behavior as long as the aspect ratios were large enough to ensure a composite wetting contact. In agreement with results obtained by Öner et al. [15], we found that the CAs remained the same as the post height was varied while the other parameters were held constant (this was checked by measuring the CAs on surfaces with identical post diameters and solid fractions, but different post heights).

In accordance with results obtained previously for larger scale post surfaces [14], the advancing CAs on the surfaces proved to be relatively independent of the surface geometry. Values between around 170 and 175° were determined, but no clear trend with post size could be established. In contrast, the receding angles showed a strong variation as the surface size scale and/or the solid fraction were varied. For the receeding angle values between 123 and 168° were measured. In general, where comparable with respect to post size, our results were on the same order of magnitude as those reported in the literature: For a surface with *s* and *d* = 8 μm, Öner obtained θ_Adv_/θ_Rec_ = 173°/134° [15], compared to 171°/126° in our case. Precise quantitative agreement cannot be expected in light of the fact that both the surface chemistry and the grid pattern (hexagonal in Öner’s versus quadratic in our case) were different.

**Figure 4:**
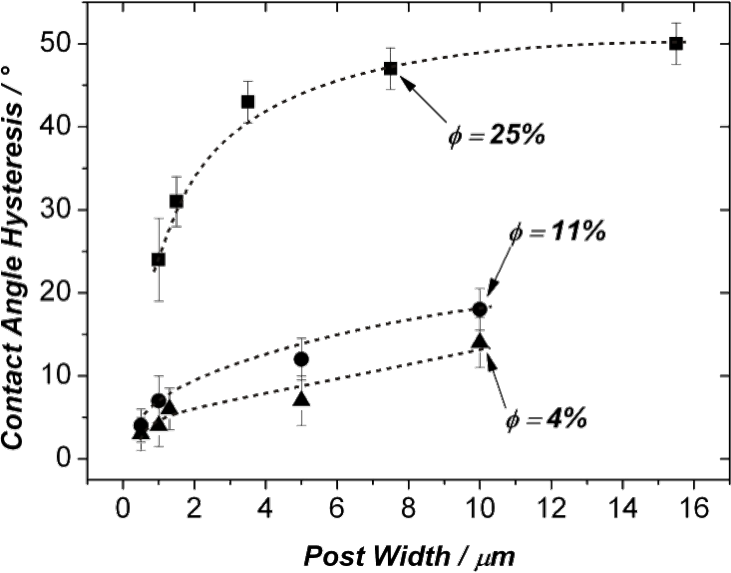
Contact angle hysteresis on fluoropolymer-coated silicon post surfaces. The solid fractions 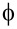 are indicated. It should be noted that the dashed lines are guides to the eye only and have no physical meaning.

Figure 4 shows the CA hysteresis Δθ = θ_Adv_ - θ_Rec_ for the three series (solid fractions of 25%, 11% and 4%) as a function of the surface size scale. Focussing on the two curves corresponding to the surfaces where 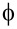 = 11 and 4%, the following is observed: Starting from post widths above 10 μm, the contact angle hysteresis decreases steadily from values of around 15° to values below 5° for the smallest posts. The latter hysteresis values corresponded to roll-off angles below 4–5° for drops of 2 μL size, which is in agreement with results obtained by Fürstner et al. for similarly sized post surfaces [25]. Simultaneously, as is apparent from Figure 4, the effect of the solid fraction on the hysteresis becomes less pronounced as smaller post widths are reached: The datapoints for *d* = 0.5 and 1 μm are clustered in a very small area in the lower left of the diagram. In Figure 4, the highest hysteresis value is found for the surface where *d* = 16 μm and 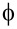 = 25%. On this substrate, Δθ was around 60°, i.e., much higher than the values observed for the surfaces characterized by the lower solid fractions. For this experimental series (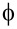 = 25%), a strong downward trend in the hysteresis is apparent below post widths of around 5 μm. However, for the post widths studied in this experimental series, the “truly” superhydrophobic regime was not reached: For a solid fraction of 25%, a minimum value of Δθ = 24° was found for a post width of 1.2 μm.

From the above experimental findings, two major trends in the data are apparent: I) A decreasing surface size scale leads to a decrease in the contact angle hysteresis. For solid fractions equal to and below values of around 10%, the CA hysteresis drops below 5° once post sizes around 1 μm are reached. For these surfaces, very low roll-off angles (below 5°) were observed. In the present work, this behavior is defined as “true” superhydrophobicity. The delimitation of the “truly superhydrophobic” from the “sticky superhydrophobic” regime is of course to a certain degree arbitrary. However, the authors are of the opinion that a surface where a 2 μL drop requires a tilting angle of 25° in order to roll off cannot be called “truly superhydrophobic” when compared to a surface where the roll-off angle is only 5°. Only on the latter surface will the experimentalist encounter typical problems such as a difficulty in depositing drops from a syringe. II) While Figure 4 immediately shows that the solid fraction cannot, as has sometimes been proposed, be the only parameter that determines the mobility of drops on a composite surface, it also indicates that the effect of the solid fraction becomes less important as the roughness features of a surface become smaller in size.

**Figure 5:**
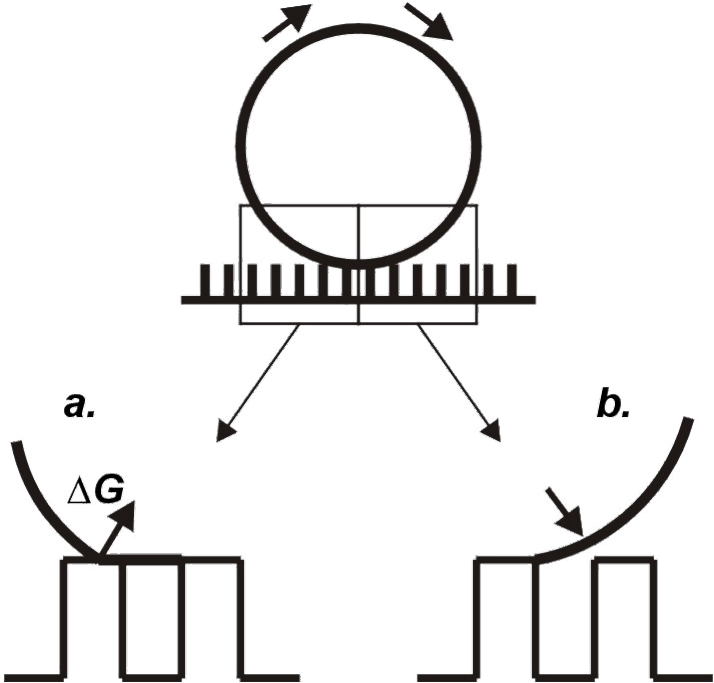
Motion of drops in the composite state: (a) Receding motion; the lifting off from a roughness feature is associated with an energy barrier Δ*G*. (b) The advancing motion entails a coming down of the meniscus onto neighboring asperities.

**Figure 6:**
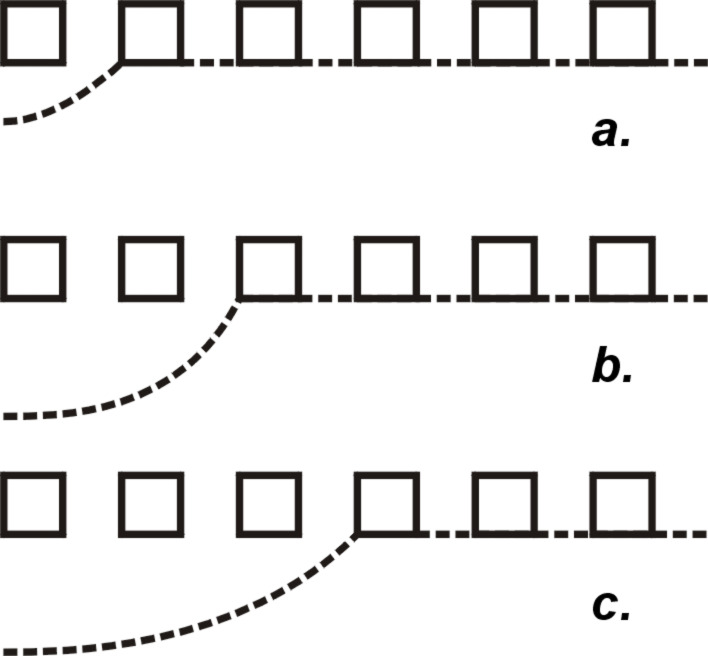
The successive dewetting of a drop from an array of roughness features is illustrated.

It has been argued in several recent publications that, as shown in Figure 5, composite drops move forward in a rolling motion [3–4,14,26–27]: The advancing meniscus comes down onto neighboring roughness features from above, while the receding meniscus has to successively dewet from the roughness features on the backside of the drop. In this model, the largest contribution to the energy barrier that opposes drop movement comes from this dewetting process. One of the findings discussed here is that even those surfaces where the solid fraction is relatively high (11%) can become superhydrophobic if the surface size scale is decreased far enough. We explain this observation along the following lines:

I) We compare the dewetting process for a surface with a large size scale with that for a surface with a smaller size scale, both with the same solid fraction and the same topology. For the surface with the larger roughness features, the receding meniscus has to dewet from fewer, but larger surface features. In contrast, for the surface with the smaller size scale, the receding meniscus has to dewet from more, but smaller surface features. The dewetting will probably not take place from all surface features at once, but successively from one roughness feature at a time over the length of the contact line in a zipping motion (as sketched in Figure 6). The height of the energy barrier for the entire dewetting process is thus determined by the energy barrier for the dewetting from an individual roughness feature. The dewetting from a smaller roughness feature is easier, and thus associated with a higher receding contact angle, because an smaller interfacial area is involved.

II) It is probable that the distortion of the contact line also plays a role [23,28–30]: On a surface with a smaller size scale, the contact line is probably more strongly distorted than on a surface with larger surface features. This distortion of the contact line will provide an additional force that facilitates drop movement.

## Conclusion

In this work, the effect of surface size scale on the behavior of drops in the composite state has been studied systematically. The experimental results show that surfaces for which the solid fraction is not too high become superhydrophobic as the size scale of the roughness features falls below around 1 μm. For larger size scales, high static contact angles may appear, but the roll-off angles remain high (>15°). It is believed that this behavior is due to the fact that, when composite drops move, the dewetting motion on the backside of the drop is the rate-limiting step. Since dewetting takes place in a zipping motion, it is easier for surfaces with smaller roughness features, and is thus associated with a higher receding and lower roll-off angle.

## Experimental

### Silicon micromachining

Using standard lithographic techniques and reactive ion etching, different patterns of shapes were transferred from a photomask into an oxide layer on 4 inch, silicon (100) wafers. The oxide acted as a masking layer in the subsequent anisotropic etching step, where the actual three-dimensional post structure was fabricated. The processed wafers were broken into individual dies and cleaned in de-ionized water, 2-propanol and acetone.

#### Surface modification

For the chemical surface modification, a benzophenone-based silane (4-(3’-chlorodimethylsilyl)propyloxybenzophenone) was first synthesized and immobilized at the micromachined surfaces according to procedures described previously [14,23–24].

A thin film of poly(3,3,4,4,5,5,6,6,7,7,8,8,9,9,10,10,10-heptadecafluorodecylacrylate) (PFA, synthesis described previously [24]) was then applied onto the surfaces by dipping the samples into a 10 mg·ml^−1^ solution of the polymer in 1,1,2-trichlorotrifluoroethane (Freon) and withdrawing them at a fixed speed of 1 mm·s^−1^. After drying, the film was exposed to UV radiation (flood exposure, λ = 265 nm, *t* = 5 min). During this irradiation step, a monomolecular polymer layer was covalently attached to the substrates. Subsequently, the surfaces were submitted to a rigorous extraction (Soxhlet extraction, *t* = 10 h, with 1,1,2-trichlorotrifluoroethane as a solvent). As has been previously shown, this procedure results in thin, surface-attached fluoropolymer films with a thickness on the order of 10 nm [24]. The fluoropolymer film completely masks the underlying surface chemistry.

#### Contact and roll-off angle measurements

The CAs were determined according to the sessile drop method using an OCA20 system from Dataphysics GmbH with de-ionized, Millipore filtered water as a test liquid. The volumes of the drops were on the order of 2 μL. For the advancing/receding measurements, liquid was added to/withdrawn from the drops at a fixed rate (0.1 μL·s^−1^) using a syringe pump. The transient behavior of the CA was recorded and found to plateau as the advancing and receding values were reached (these values remained constant also if larger/smaller drop sizes were reached). Each angle was measured multiple times, resulting in an average value with a standard deviation in the range of 2°. For thin films of PFA attached to polished silicon slides, we determined the following CAs: θ*_Adv _/*θ*_Rec_* = 120° ± 2°/106° ± 3°. We do not give static CAs; as has often been pointed out, the static CA is a relatively arbitrary quantity that may assume more or less any value between the receding and the advancing CA. It depends, among other things, on how the respective drop has been deposited or for how long it has been left to evaporate. Finally, the roll-off angles were determined by placing 2 μL drops on the substrates. The samples were then progressively tilted and the angle at which drop movement set in was recorded. Smaller drops required larger tilting angles in order to roll off; a constant drop size of 2 μL was chosen in order to be able to compare the measurements on the different surfaces and because this drop size has a large practical relevance (this drop size is often used in measurements according to the sessile drop method).
